# Identification of candidate microRNA biomarkers of endometriosis in different bodily fluids

**DOI:** 10.1038/s41598-026-37277-5

**Published:** 2026-01-25

**Authors:** Shiqing Lyu, Qiutong Li, Zhiyue Gu, Hailan Yan, Xinyue Tang, Yi Dai, Xiaoyan Li, Yushi Wu, Chenyu Zhang, Yiyao Xu, Yuanyuan Li, Yao Hu, Wing Hing Wong, Yanqin Yu, Shen Lu, Farideh Z. Bischoff, Jinhua Leng, Jinghua Shi

**Affiliations:** 1 Department of Obstetrics and Gynecology, Peking Union Medical College Hospital, Chinese Academy of Medical Science & Peking Union Medical College, National Clinical Research Center for Obstetrics & Gynecologic Diseases, No. 1 Shuaifuyuan, Dongcheng District, Beijing, 100730 China; 2 Hangzhou Yizhen Medical Laboratory Co., Ltd., Hangzhou, China; 3 HerAnova Lifesciences, Boston, MA United States of America

**Keywords:** Endometriosis, MicroRNA, Bodily fluids, Biomarker, Noninvasive diagnosis, Biomarkers, Computational biology and bioinformatics, Diseases, Medical research, Molecular biology

## Abstract

Endometriosis is a chronic estrogen-dependent disorder affecting up to 10% of women of reproductive age, and the absence of reliable noninvasive diagnostic tools contributes to delayed diagnosis and disease progression. To identify potential biomarkers, we profiled miRNA expression in serum, saliva, and vaginal mucus from 20 women (10 with endometriosis and 10 controls) using next-generation sequencing. Differentially expressed miRNAs were identified, and their predicted targets underwent Gene Ontology and KEGG pathway enrichment analyses. Serum proteomics by data-independent acquisition LC–MS/MS was integrated with miRNA data to construct potential miRNA–protein interaction networks. Distinct miRNA profiles were observed across the three bodily fluids, with serum showing the most abundant miRNAs and saliva the lowest. Thirteen, three, and six differentially expressed miRNAs were detected in serum, saliva, and vaginal mucus, respectively. Enrichment analysis implicated apoptosis, Wnt signaling, autophagy, and cellular senescence. Integrated analysis revealed 59 upregulated serum proteins targeted by dysregulated miRNAs, including WNK2, CD44, USP15, GNAI3, HUWE1, and NRAS. ROC analysis suggested that serum miR-200a-3p and miR-200b-3p, may have potential utility as noninvasive biomarkers for the diagnosis and monitoring of endometriosis, pending further validation.

**Supplementary Information:**

The online version contains supplementary material available at 10.1038/s41598-026-37277-5.

## Introduction

 Endometriosis is a common gynecological condition that affects approximately 10% of women of reproductive age^1^. Clinical presentations vary widely, ranging from pelvic pain, infertility, and pelvic masses to asymptomatic cases. Although initial suspicion often arises from symptoms, physical examination, and imaging, none of these methods can definitively diagnose the disease^2^. As a result, confirmation currently relies on invasive laparoscopy, contributing to a substantial diagnostic delay—typically between 5 and 12 years from symptom onset—during which patients often consult multiple physicians^1^.

Therefore, there is a pressing need to identify reliable, noninvasive biomarkers to aid in early diagnosis and disease monitoring. Numerous studies related to this topic have been published, but currently no noninvasive diagnostic test could replace laparoscopy for diagnosis of endometriosis in clinic^3–6^.

MicroRNAs (miRNAs) are 19–25 nucleotides long, non-coding RNAs that regulate gene expression post transcriptionally^7^. MiRNAs are known to be related to many disease statuses, such as malignancy, infectious diseases and autoimmune diseases^8–10^, and evidence suggests their involvement in the pathogenesis of endometriosis^11,12^. Importantly, miRNAs are relatively stable molecules and can be detected in various bodily fluids^13–15^, underpinning their potential utility as non-invasive diagnostic biomarkers. Recently, a French research team established a saliva-based diagnostic panel consisting of 109 miRNAs analyzed with artificial intelligence (AI) modeling. In validation testing, the panel achieved sensitivity, specificity, and an area-under-the-curve (AUC) exceeding 95%^16^. While these results are encouraging, such approaches face challenges for routine clinical implementation.

Several limitations hinder the clinical translation of current miRNA-based approaches. The requirement for large miRNA panels (e.g., 109 miRNAs) renders comprehensive profiling technically complex and cost-prohibitive for widespread clinical application, particularly when based on next-generation sequencing. In addition, most studies focus on a single biofluid --- most commonly blood or saliva --- which may limit the robustness and reproducibility of the findings^17^. Given the anatomical proximity, the vaginal mucus may present an alteration in biomarkers influenced by endometriosis, but this potential relationship remains poorly investigated. To date, only one proteomic study has examined cervical mucus in endometriosis identified several dysregulated proteins^18^, while the miRNA profile of vaginal mucus in this disease context remains unknown. Furthermore, hormonal fluctuations across the menstrual cycle could confound circulating miRNA profiling in women of reproductive age.

In this study, we aimed to profile and compare miRNA expression patterns across three types of samples—serum, saliva, and vaginal mucus. Samples collected from individuals with endometriosis and from a control group without endometriosis. By providing the first integrated miRNA profile from these distinct sources, this approach may offer new insights into the systemic and local molecular changes underlying the disease and may enhance future noninvasive diagnostics.

## Results

### Clinical characteristics of patients

The clinical characteristics of the patients from the sequencing cohort are presented in Table 1. No significant differences in age, body mass index (BMI), menstrual cycle or parity were found between two groups. The CA125 level was greater in ENDO group than in control group numerically. Six of patients in ENDO group suffered from dysmenorrhea, while only 1 patient in control group experienced dysmenorrhea. The numerical rating scale (NRS) score was significantly higher in ENDO group than in control group. All the patients in ENDO group had rASRM stages III-IV.

**Table 1:** Clinical characteristics of the participants in the sequencing cohort.

Characteristics	ENDO (*N* = 10)	Control (*N* = 10)	*P*-value
Age (years), mean (SD)	33.0 (5.3)	36.0 (5.3)	0.22
BMI (kg/m^2^), mean (SD)	21.9 (1.6)	23.3 (3.4)	0.26
Menstrual cycle (days), median [IQR]	29.0 [26.5, 30.0]	30.0 [30.0, 32.0]	0.07
Parity, median [IQR]	0 [0.0, 1.8]	0 [0.0, 1.8]	1.00
CA125 (U/ml), median [IQR]	38.1 [28.9, 59.3]	14.8 [13.0, 26.6]	0.08
Menstrual cycle, n (%)			-
Proliferative phase	5 (50.0)	5 (50.0)	-
Secretory phase	5 (50.0)	5 (50.0)	-
Dysmenorrhea, n (%)	6 (60.0)	1 (10.0)	0.06
NRS, median [IQR]	2.5 [0.0, 5.0]	0 [0.0, 0.0]	0.03
Severity of endometriosis (rASRM), n (%)			-
Moderate (III)	5 (50.0)	-	-
Severe (IV)	5 (50.0)	-	-

### Overview of miRNA expression profiles in serum, saliva and vaginal mucus

**Fig. 1:**
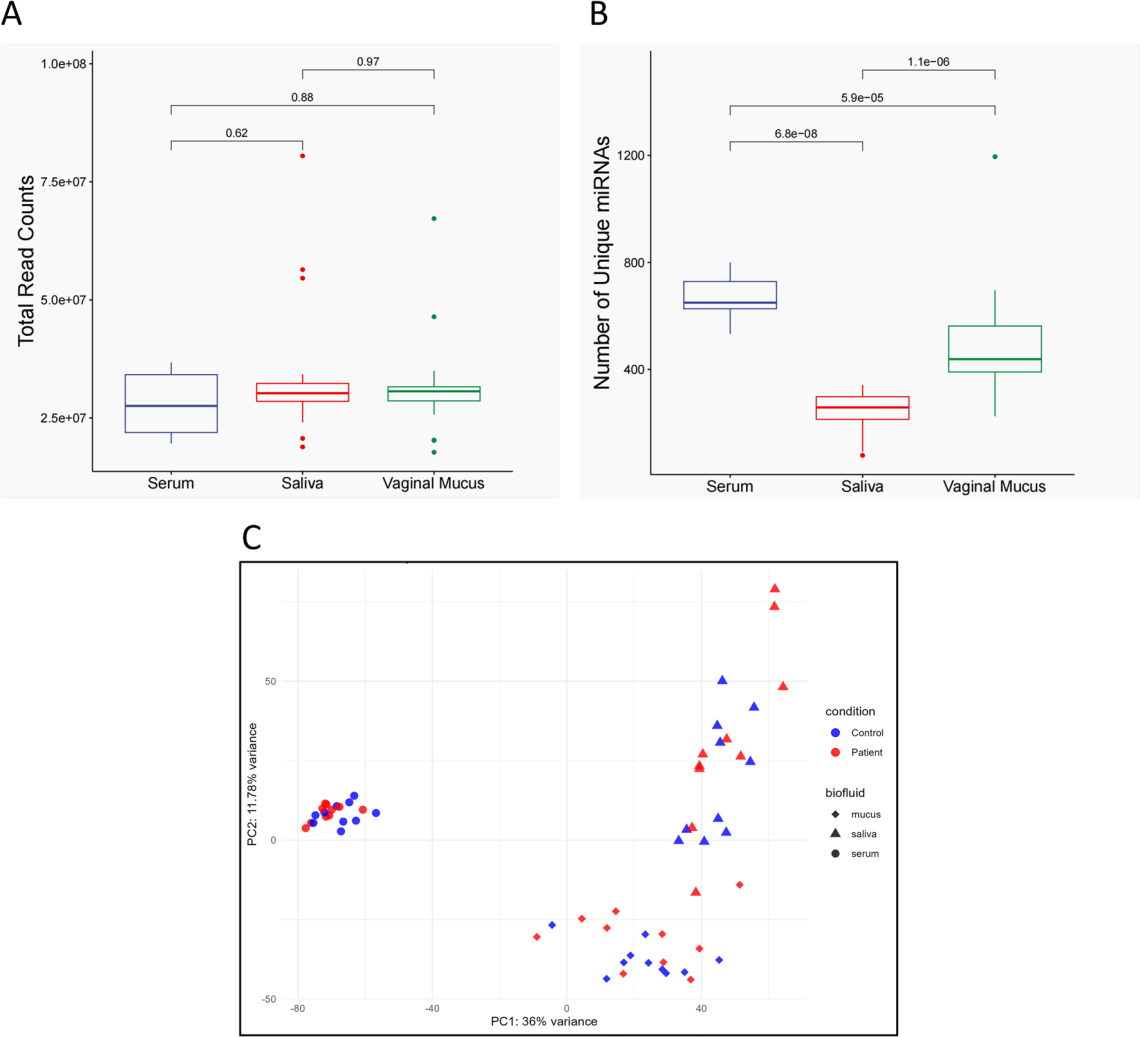
Overview of miRNA expression profiles in serum, saliva and vaginal mucus. (**A**), Box plot illustrating the total number of raw reads for each sample type. (**B**), Box plot showing the number of unique miRNAs detected in each sample type, defined as miRNAs with a read count greater than 0. (**C**), PCA of miRNA expression profiles.

We successfully obtained small RNA sequencing data from all samples. The distributions of total reads and the number of mapped miRNAs across different sample types are summarized in Fig. 1. Detailed read counts, quality control and miRNA detection results for each sample are shown in supplementary Figures S1, S2 and Table S1. No significant differences were observed in total input reads among serum, saliva, and vaginal mucus, indicating comparable sequencing depth across sample types. In contrast, the number of detected miRNAs differed significantly, with serum showing the highest number of mapped miRNAs and saliva showing the lowest.

Principal component analysis (PCA) based on global miRNA expression profiles revealed a clear separation of serum samples from saliva and vaginal mucus samples along the PC1 axis, indicating distinct miRNA expression patterns among the different biological matrices.

To assess the potential confounding effect of the menstrual cycle on miRNA biomarkers, we performed a likelihood ratio test to compare the full model (including the interaction between disease condition and menstrual phases) against a reduced model containing only the main effects. The analysis revealed that the menstrual cycle phases had a limited overall impact. Specifically, significant phase effects were detected for only two miRNAs (hsa-miR-122-3p and hsa-miR-122-5p) exclusively in serum samples, whereas no significant effects were detected for the other two sample types analyzed. These findings indicates that menstrual cycle variation introduces minimal bias to the miRNA profiles in this study.

### Identification of differentially expressed miRNAs and their target genes in different bodily fluids

**Fig. 2:**
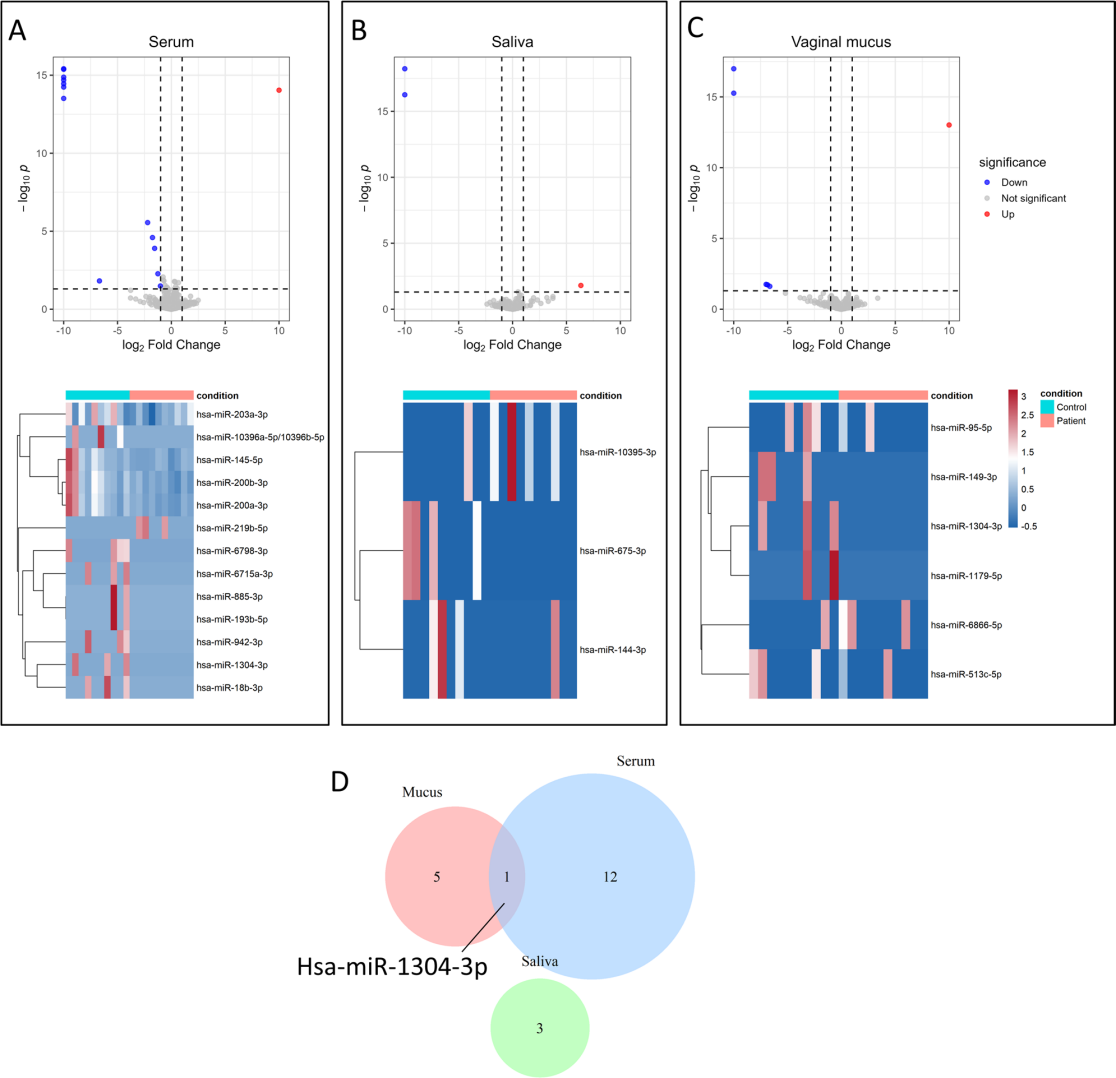
DEMs in serum, saliva and vaginal mucus. (**A**,** B** and** C**), Volcano plots and heatmaps showing DEMs in three types of bodily fluids. (**D**), Venn diagram demonstrating that only hsa-miR-1304-3p is differentially expressed in both serum and mucus. No common DEMs for three types of fluids were identified.

Using a nominal p-value < 0.05, |log2FoldChange| > 1 and base mean counts > 10 as the cutoff values, we identified 13, 3 and 6 differentially expressed miRNAs (DEMs) in serum, saliva and vaginal mucus respectively (Fig. 2) (Table 2).

Furthermore, as shown by Venn Diagram, no common DEMs across all bodily fluids were identified. This observation suggests that the miRNA signature may be distinct between different fluid types, potentially reflecting their unique biological origins and functions. MiR-1304-3p was the only miRNA that was differentially expressed between both serum and mucus. However, this miRNA was detected in fewer than half of the cases (Fig. 2).

**Table 2:** DEMs in serum, saliva and vaginal mucus.

Bodily fluid	Upregulated	Downregulated
Serum	hsa-miR-219b-5p	hsa-miR-942-3p, hsa-miR-1304-3p, hsa-miR-885-3p, hsa-miR-193b-5p, hsa-miR-10396a-5p/10396b-5p, hsa-miR-6715a-3p, hsa-miR-18b-3p, hsa-miR-200b-3p, hsa-miR-200a-3p, hsa-miR-145-5p, hsa-miR-203a-3p, hsa-miR-6798-3p
Saliva	hsa-miR-10395-3p	hsa-miR-675-3p, hsa-miR-144-3p
Vaginal mucus	hsa-miR-6866-5p	hsa-miR-1304-3p, hsa-miR-1179-5p, hsa-miR-513c-5p, hsa-miR-95-5p, hsa-miR-149-3p

To gain insight into the potential roles of the differentially expressed miRNAs, we performed a comprehensive prediction of their target genes. Using a combination of validated miRNA target databases (e.g., Mirecords, Mirtarbase and Tarbase), we identified a total of 8342 unique putative target genes in serum, 478 in saliva and 2808 in vaginal mucus.

As expected, given that miRNAs typically function by repressing target gene expression, a significant proportion of the targets of downregulated miRNAs are frequently associated with activated pathways.

### GO enrichment analysis and KEGG pathway analysis of target genes in endometriosis

**Fig. 3:**
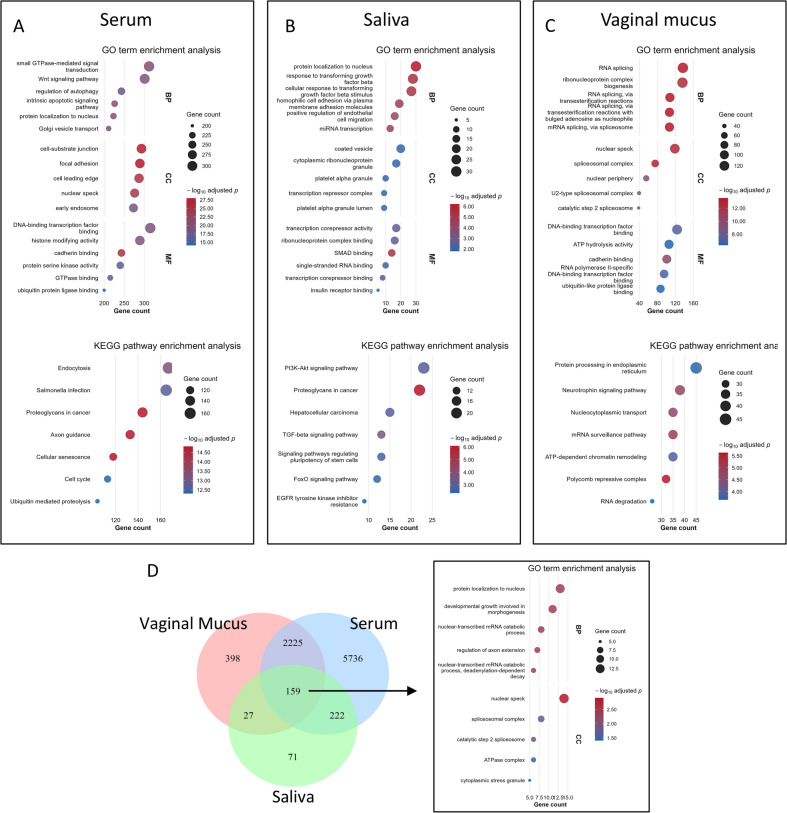
GO enrichment analysis and KEGG signaling pathway analysis of target genes. (**A**,** B** and** C**), GO and KEGG analysis of target genes in the serum, saliva and vaginal mucus respectively^19,20^.(** D**), Venn diagram of common target genes between three types of fluids and the GO analysis of common genes.

We identified the top 10 significant Gene Ontology (GO) and signaling pathways with the criterion of a false discovery rate (FDR) < 0.05 using predicted target genes of DEMs. In serum, major genes were related to intrinsic apoptotic signaling pathway, Wnt signaling pathway and regulation of autophagy. Kyoto Encyclopedia of Genes and Genomes (KEGG) pathway analysis revealed that DEMs were enriched mainly in cellular senescence, axon guidance and proteoglycans in cancer. In saliva, BP was enriched mainly in cellular response to TGF-β, homophilic cell adhesion via plasma membrane adhesion molecules and positive regulation of endothelial cell migration. KEGG analysis revealed that most related pathways were proteoglycans in cancer, TGF-β signaling pathway and PI3K-Akt signaling pathway. In vaginal mucus, DEMs were mostly related to RNA splicing and ribonucleoprotein complex biogenesis. Most related signaling pathways included polycomb repressive complex, mRNA surveillance pathway and neurotrophin signaling pathway (Fig. 3).

In an effort to explore potential common disease pathways, we identified 159 shared target genes of miRNAs across the three bodily fluids. Functional enrichment analysis revealed that these genes were enriched primarily in the biological processes related to protein localization to nucleus, developmental growth involved in morphogenesis and regulation of axon extension (Fig. 3).

## Combined proteome sequencing for potential biomarker identification

**Fig. 4:**
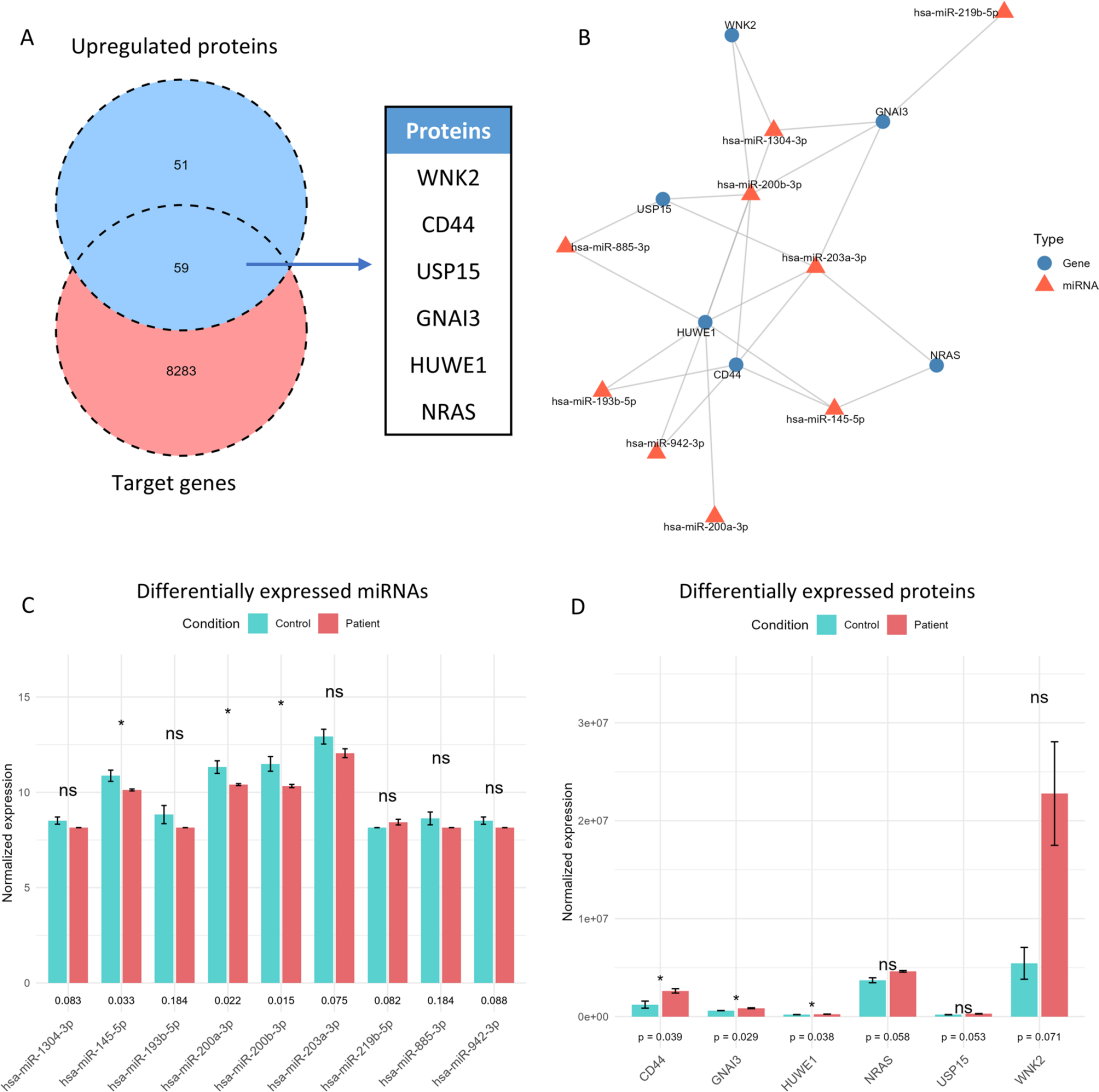
The combination of target genes and upregulated proteins to identify potential biomarkers of endometriosis. (**A**), Six proteins were selected from the intersection of upregulated proteins and target genes of miRNAs in serum. (**B**), Interaction of target genes and miRNAs. (**C**,** D**), T-test of normalized expression of selected DEMs and DEPs.

Among the three bodily fluids analyzed, serum presented the highest abundance of miRNAs. To gain deeper insight into the pathological mechanisms and identify potential biomarkers, we performed a proteomic analysis of serum from patients with endometriosis and healthy controls. This approach identified 59 upregulated differentially expressed proteins (DEPs) that were predicted targets of DEMs. Among these upregulated proteins, several were found to be associated with key biological processes and signaling pathways such as the Wnt signaling pathway, apoptosis, autophagy, and cellular senescence. These proteins include WNK2, CD44, USP15, GNAI3, HUWE1 and NRAS. Furthermore, DEMs that targeted these proteins were miR-1304-3p, miR-145-5p, miR-193b-5p, miR-200a-3p, miR-200b-3p, miR-203a-3p, miR-219b-5p, miR-885-3p and miR-942-3p (Fig. 4).

### Validation of serum miRNAs candidates by qPCR

**Fig. 5:**
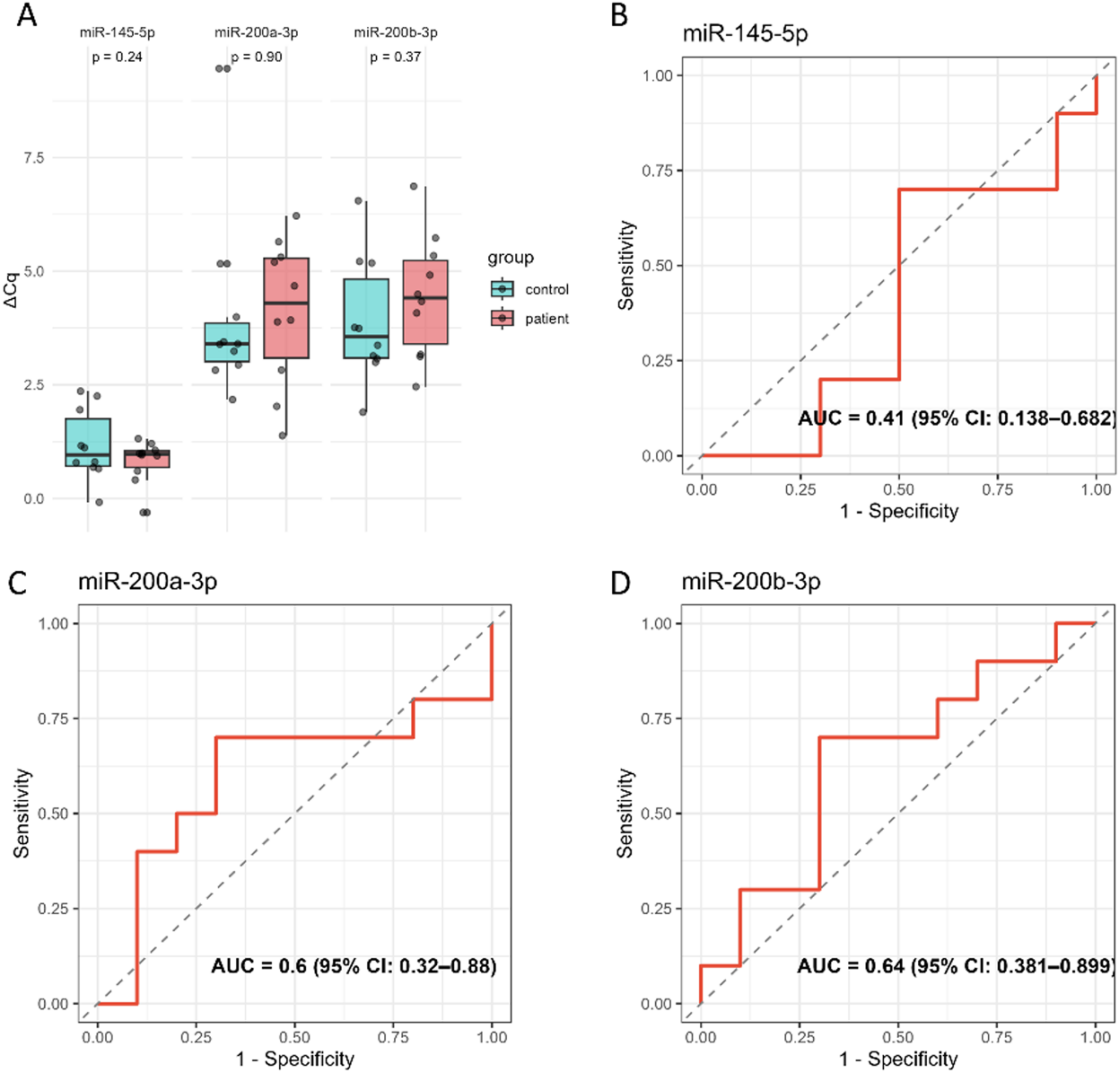
QPCR validation of selected miRNAs in serum. (**A**), Expression levels of candidate miRNAs measured by qPCR. The expression trends for miR-200a-3p and miR-200b-3p were consistent with the NGS sequencing results. (**B**-**D**), ROC curves evaluating the diagnostic potential of the respective miRNAs based on qPCR data.

To further validate the miRNA candidates identified through sequencing, we performed qPCR analysis on serum samples from the same cohort of 20 patients. The results showed that miR-200a-3p and miR-200b-3p exhibited AUC values of 0.60 (95% CI: 0.32–0.88) and 0.64 (95% CI: 0.38–0.90), respectively, suggesting a moderate discriminatory ability of these miRNAs for endometriosis. Leave-one-out cross-validation (LOOCV) was performed to assess model stability, and the cross-validated AUC values were identical to those obtained from the full dataset. The relatively wide confidence intervals reflect the uncertainty in point estimates, likely due to the limited sample size, emphasizing the need for validation in larger cohorts. Nevertheless, the consistent direction of expression changes observed in both NGS and qPCR analyses supports the potential relevance of these miRNAs (Fig 5).

## Discussion

In this study, we thoroughly analyzed the miRNA expression profiles of three bodily fluids that are most commonly and easily obtained under gynecological conditions --- serum, saliva and vaginal mucus. This study employed a standardized protocol to collect and analyze bodily fluids simultaneously. By using NGS, our analysis revealed that miRNAs exhibit fluid-specific expression to a great extent; nevertheless, their predicted target genes intersect and converge on shared biological pathways. Furthermore, by integrating miRNA profiling from peripheral blood with proteomic data, we identified several miRNAs and their target genes with potential biological significance in endometriosis.

To our knowledge, this is the first prospective study to characterize and compare miRNA profiles across three types of bodily fluids. Notably, this study is the first to analyze miRNA expression in vaginal mucus, which we found containing greater amounts of miRNAs detected than saliva does. Vaginal mucus is routinely collected during gynecological visits and can be easily obtained through standard examinations such as bimanual pelvic exams—a fundamental procedure in gynecological practice. While higher miRNA abundance alone does not necessarily translate into superior diagnostic performance, this finding suggests that vaginal mucus may represent a technically feasible and underexplored biological matrix for miRNA-based biomarker discovery. Very few studies have explored biomarkers for endometriosis in vaginal secretions. To date, only one study has investigated the proteomic profile of cervical mucus in endometriosis patients​^18^, yet no consistently upregulated proteins have been identified compared with our research. Given that miRNA profiling in vaginal mucus could offer a novel, noninvasive approach for diagnosing endometriosis, further studies focusing on this fluid are warranted.

Despite more than 90% similarity in miRNA expression profiles between serum and saliva^14^, no common differentially expressed miRNAs were detected. Only miR-1304-3p was found in both serum and vaginal mucus; this gene was previously reported to be upregulated in ectopic endometrium and may be related to endometrial receptivity^21^. We also discovered that common target genes of three types of bodily fluids may reflect similar biological processes such as developmental growth involved in morphogenesis, regulation of axon extension and regulation of extension of cell growth. This may suggest that while different bodily fluids reflect their own unique biological processes, they may also share a set of common, disease-driven pathological processes.

Several previous studies have reported saliva-based miRNA panels as potential noninvasive diagnostic tools for endometriosis by using AI algorithms^16,22^. In the present study, however, the number of detected miRNAs and differentially expressed miRNAs identified in saliva was substantially lower than that observed in serum. This discrepancy may be attributable to differences in cohort size, sequencing depth, and disease heterogeneity, as well as the inherently lower and more variable RNA abundance in saliva compared with serum. Our results suggest that saliva-derived miRNAs may serve as complementary biomarkers rather than standalone diagnostic indicators and need further investigation.

Blood is the most commonly used bodily fluid for noninvasive diagnosis and has been the most extensively studied method in endometriosis^17^. Our findings concerning miR-200a-3p are consistent with those of previous studies in blood^23^. MiR-200b-3p was reported to be upregulated in deep infiltrated endometriosis^24^. MiR-200 family is known to play a crucial role in epithelial-mesenchymal transition^25^. MiR-200a-3p is one of the miRNAs that targets PTEN in endometriosis and ovarian cancer and miR-200b targets PTEN in metastatic prostate cancer^24^. Some of the miRNAs discovered in our study, such as miR-193b-5p, miR-145-5p, miR-203a-3p and miR-144-3p, was mentioned to be dysregulated in eutopic or ectopic endometrium or uterine fluid^26–32^. They may function in ferroptosis, migration and proliferation and lead to the pathological mechanisms of endometriosis. Furthermore, we identified several previously unreported miRNAs dysregulated in endometriosis, including miR-219b-5p, miR-942-3p, miR-885-3p, miR-10395-3p, miR-10396a-5p/10396b-5p, miR-6715a-3p, miR-18b-3p, miR-6798-3p, miR-675-3p, miR-6866-5p, miR-1179-5p, miR-513c-5p, miR-95-5p and miR-149-3p. Notably, literature suggests that some of these miRNAs (e.g., miR-942-3p, miR-885-3p) are involved in critical pathways such as ERK and Akt-NFκB signaling pathways^33–35^, which are also relevant to endometriosis pathogenesis. MiR-149-3p may promote inflammation and malignancy in gastroenterological diseases^36^. Other miRNAs, such as miR-18b-3p and miR-6798-3p, have been associated with fibrosis and apoptosis^37,38^, which are key processes in the establishment of endometriotic lesions. Among these miRNAs, miR-200a-3p and miR-200b-3p presents discriminative ability for endometriosis. However, given the limited sample size and absence of external validation, this finding requires confirmation in larger, independent cohorts.

By integrating our miRNA findings with peripheral blood proteomics, we identified 59 proteins with potential pathological and diagnostic relevance. Notable candidates include WNK2, CD44, USP15, GNAI3, HUWE1, and NRAS, which are involved in pathways such as Wnt signaling, apoptosis, autophagy, and cellular senescence—processes strongly associated with endometriosis. CD44, a known Wnt/β-catenin target, may facilitate ectopic endometrial growth^39^. Altered expression of CD44 has been observed in endometriosis and is implicated in the dysregulation of cell adhesion processes^40^. NRAS mutations have been linked to gynecological cancers including ovarian endometrioid cancer^41,42^. WNK2, although primarily studied in neural contexts, acts as a tumor suppressor and positively regulates Wnt/β-catenin signaling^43^. USP15 modulates key pathways, including TGF-β and NF-κB pathways, through deubiquitination^44^, and GNAI3 has been associated with tumor suppression and inflammation regulation^45–47^. HUWE1, a multifunctional E3 ligase, influences cellular growth, death, and inflammasome activation^48^. These proteins highlight convergent mechanisms that may underlie endometriosis pathogenesis, warranting functional validation in future studies.

Regarding vaginal mucus, a prior study investigated the proteomic profile of cervical mucus in endometriosis and reported elevated levels of proteins, including PIGR, NGAL, TIMP1, FBLN1, A1AG2 and CO3^18^. However, none of these proteins were predicted targets of the dysregulated miRNAs identified in our dataset. In the case of saliva, proteomic studies related to endometriosis remain scarce, with limited evidence available in the current literature.

In this study, we initially hypothesized that miRNA profiles in bodily fluids would be significantly influenced by the menstrual phase, particularly in vaginal mucus due to their direct anatomical connection to female reproductive tract. However, our analysis revealed that menstrual phase had a minimal effect on miRNA expression across the fluids examined. Only miR-122-3p and miR-122-5p showed potential phase-related variation. This finding aligns with previous studies suggesting that circulating miRNAs expression is largely stable throughout the menstrual phase^49–51^.

Several limitations of this study should be acknowledged. First, the relatively small sample size, consisting of 10 patients with endometriosis and 10 controls, limited the statistical power of the analyses. The use of p-value instead of FDR may also have increased the risk of false-positive findings. Accordingly, the results should be interpreted with caution and regarded as exploratory in nature. Internal and external validation in larger, independent cohorts is required to confirm the robustness and reproducibility of the identified miRNA signatures.

Second, only patients with stage III–IV endometriosis were included, which may limit the generalizability of the findings to individuals with early-stage disease. In addition, the control group consisted exclusively of patients with ovarian teratomas. While this design allowed for clinical comparability, it may not fully represent the broader non-endometriosis population. MiRNA expression profiles may vary across disease stages and different gynecological conditions; therefore, future studies incorporating a wider spectrum of endometriosis severity as well as healthy controls are warranted.

Third, although efforts were made to standardize the sample collection procedures and all participants were recruited from the northern region of China, detailed lifestyle-related factors—such as smoking status, dietary habits, and other environmental exposures—were not systematically collected. As a result, their potential influence on circulating and mucosal miRNA profiles could not be evaluated.

Furthermore, our study is a cross-sectional study. Postoperative samples or repeated measurements over time were not available, precluding assessment of dynamic changes in miRNA expression in response to surgical intervention or disease progression, which is a direction for future researches.

Finally, it should be noted that miRNA profiles derived from biofluids such as serum, saliva, and vaginal mucus may not directly reflect molecular alterations within endometriotic lesions themselves. Enriched pathways and regulatory networks identified from these samples may represent systemic inflammatory responses or secondary physiological changes rather than lesion-specific mechanisms. Therefore, caution is warranted when extrapolating biofluid-based miRNA signatures to local disease pathology. Further experimental validation on hypothesis of pathogenesis is warranted.

By pioneering the integration of multi-fluids miRNA profiling and serum multi-omics in endometriosis, this study delivers a valuable database and identifies novel biomarker candidates. Integrated pathway analysis offers fresh perspectives on disease mechanisms, paving the way for future research into improved diagnostic method and therapies.

## Methods

### Study design and study population

Participants in this cross-sectional exploratory sub analysis were drawn from an ongoing study designed to develop noninvasive diagnostic methods for endometriosis. All subjects were prospectively recruited at Peking Union Medical College Hospital, a tertiary-care center in Beijing, China. The trial was registered at the Chinese Clinical Trial Registry (ChiCTR2400093810) on 2024-12-12, and the ethics approval were obtained by the Ethics Committee of Peking Union Medical College Hospital on 2025-08-18 in accordance with the Declaration of Helsinki, approval number I-25PJ1885. All participants signed written informed consent.

The inclusion criteria were as follows:

1. Aged between 18 and 50 years (> 18 and < 50 years of age).
2. With a history of sexual activity.
3. Written informed consent was provided and all study-related questionnaires concerning symptoms and quality of life were completed.
4. Scheduled for surgery for suspected endometriosis or other benign gynecological diseases.
5. Sampled during a non-menstrual phase.
6. No hormonal treatment was given within 3 months prior to enrollment.

Patients with infection, malignancy, autoimmune disease or pregnancy were excluded. The participants’ demographic data and full medical history were recorded preoperatively. Menstrual cycle phase was defined as proliferative (Day 0–14) or secretory (Day 15–35), and was determined by pathology of endometrium or blood progesterone levels. The enrollment and sample collection were performed one day before surgery. Endometriosis was diagnosed by direct visualization of endometriotic lesions in the pelvic cavity during surgery and histological confirmation. Laparoscopy was performed by several surgical teams, all of which included a senior gynecological surgeon with more than 10 years of experience in the diagnosis and management of endometriosis and board members of the *Committee of Endometriosis*,* Obstetrics and Gynecology Branch*,* Chinese Medical Doctor Association*. The revised American Society of Reproductive Medicine (rASRM) classification system was used to categorize the severity of endometriosis.

Twenty patients were enrolled for NGS of miRNAs. Only patients with moderate-severe (rASRM stage III-IV) endometriosis were included in the ENDO group, and participants with benign teratoma were selected as control group. Each group contained 10 participants, with half in the proliferative phase and half in the secretory phase.

### Sample collection

For each patient, serum, saliva and vaginal mucus were collected via standardized procedures to ensure consistency.

Serum: Peripheral blood samples were collected into 10 ml collection tubes (Becton, Dickinson and Company, USA). Within 2 h, serum was separFated by centrifugation at 1900 × g for 10 min at 4℃. Supernatant was collected and further centrifuged at 16,000 × g for 10 min at 4℃. Serum was stored in aliquots at -80 °C.

Saliva: Participants were instructed to refrain from eating, drinking (including water), smoking, chewing gum, or using oral hygiene products for at least 30 min prior to collection. The saliva was expectorated into a collection tube until the liquid reached the 1 mL mark. Once 1 mL of saliva was collected, a pre-filled preservation buffer was carefully poured into the tube containing the saliva. Samples were stored at-80 °C for long-term preservation.

Vaginal mucus: Vaginal secretion samples were collected via sterile swabs. Sampling was performed by gently inserting the swab into the posterior vaginal fornix, and rotating it lightly for approximately 20 s. The swab was then inserted into a sample collection tube containing a DNA/RNA shield (Zymo Research, USA). Samples were stored at − 80 °C for long-term preservation.

### Next-generation sequencing

Total RNA was extracted from 300 µl of serum using the Plasma/Serum Circulating and Exosomal RNA Purification Kit (Norgen Biotek, Canada) according to the manufacturer’s instructions. To assess RNA extraction efficiency, an exogenous spike-in control was added during the extraction process. The spike-in assay indicated no significant differences in miRNA extraction efficiency among the different sample types (Supplementary Table S3). The isolated total RNA was stored at − 80 °C for subsequent experiments.

NGS involves steps such as library construction, quantification, sequencing, and data analysis. First, the isolated RNA was ligated with 3′ adapters by first denaturing at 70 °C for 2 min, followed by incubation with T4 RNA Ligase 2 (New England Biolabs, USA) at 16 °C for ≥ 8 h. Subsequently, 5′ adapters were ligated using T4 RNA Ligase 1 (New England Biolabs, USA) at 37 °C for 60 min. The adapter-ligated RNA was reverse transcribed in a thermocycler using SuperScript II Reverse Transcriptase (Thermo Fisher Scientific, USA) under the following conditions: 50 °C for 60 min, followed by enzyme inactivation at 80 °C for 10 min. Following cDNA synthesis, library preparation was performed using Phusion High-Fidelity DNA Polymerase (New England Biolabs, USA) in accordance with the manufacturer’s protocol. The final libraries were sequenced via high-throughput sequencing to profile miRNA expression via a 50 bp single-end setting.

Raw fastq files were preprocessed using the miRge3 pipeline^52^ which included adapter trimming via an integrated Cutadapt wrapper^53^. The resulting trimmed reads were then aligned to the miRBase reference database^54^ using Bowtie^55^, also wrapped within miRge3 and run with default parameters. Subsequently, miRge3 generated a count matrix summarizing miRNA abundances across all samples. This count matrix served as input for differential expression analysis using the DESeq2 package in R^56^. DESeq2 applies median-of-ratios normalization to account for differences in sequencing depth and library composition across samples and employs a negative binomial model to identify miRNAs with statistically significant expression differences between patient and control groups.

### Proteomic analysis

Serum samples were subjected to high-abundance protein depletion using magnetic beads. The resulting peptides were digested into peptides, which were subsequently desalted using a peptide desalting spin column (89851, Thermo Fisher Scientific). Liquid chromatography-tandem mass spectrometry (LC-MS/MS) was performed on a Thermo Scientific Vanquish Neo UHPLC system coupled to an Astral mass spectrometer operated in data-independent acquisition (DIA) mode. The acquired DIA data were processed with DIA-NN (v1.9.2) using the UniProtKB *Homo sapiens* database^57^. Differentially expressed proteins were identified based on a Student’s t-test (*p* < 0.05) and a fold change > 1.2 or < 1/1.2.

For integrative miRNA–protein analysis, upregulated proteins were intersected with predicted target genes of differentially expressed serum miRNAs. This approach aimed to identify candidates showing directional consistency between miRNA dysregulation and protein abundance.

### MiRNA reverse transcription and qPCR analysis

Total RNA was subjected to reverse transcription using miRNA 1st strand synthesis kit (Accurate Biotechnology, Hunan, China) according to the manufacturer’s instructions. Equal volumes (3.75ul) of total RNA from each sample were used for reverse transcription to generate cDNA, ensuring comparability across samples.

Next, we identified miRNA as the best endogenous control in our cohort. Based on the sequencing data, candidate endogenous control miRNAs were initially screened using the HeraNorm software^58^. These candidates were subsequently evaluated by RT–qPCR across all samples. The stability of candidate reference miRNAs was comprehensively assessed by integrating RefFinder with equivalence testing using the Two-One-Sided Test (TOST) approach^59–61^. Through this combined strategy, miR-423-3p was identified as the optimal reference miRNA, showing stable expression and no significant difference between the patient and control groups, and was therefore used as the endogenous control for normalization.

Then, target miRNAs were quantified by RT-qPCR. CDNA was diluted 10-fold prior to amplification. QPCR was performed using SYBR Green Premix Pro Taq HS qPCR Kit II (Accurate Biotechnology, Hunan, China) following the manufacturer’s protocol. All reactions were carried out under standardized cycling conditions, and each sample was analyzed in technical replicates. Relative miRNA expression levels were calculated using the ΔCq method with miR-423-3p as the endogenous control.

### Statistical analysis

Given the exploratory nature of this study, which aims to generate hypotheses and capture a broad spectrum of potentially dysregulated miRNAs for further investigation, we adopted a lenient significance threshold.​​ Differentially expressed miRNAs were identified on the basis of a nominal p-value < 0.05.

Functional enrichment analysis of target genes for the prioritized miRNAs was performed for GO terms and KEGG pathways using the ClusterProfiler package^62^ using R software (version 4.4.2). A hypergeometric test was used, and enriched terms with a p-value < 0.05 were considered significant.

The data are expressed as mean ± standard deviations (SDs) or medians (interquartile ranges). Demographic and clinical characteristics were compared using the Student’s t-test for normally distributed data. The Mann-Whitney U test was used for small sample sets or if normal distribution of the data was not confirmed with the Shapiro-Wilk test for continuous variables. Fisher’s exact test was used for categorical variables.

## Supplementary Information

Below is the link to the electronic supplementary material.

Supplementary Material 1

Supplementary Material 2

Supplementary Material 3

Supplementary Material 4

## Data Availability

The miRNA sequencing datasets generated during the current study are available from the China National Center for Bioinformation with the accession number [HRA014397] (https:/ngdc.cncb.ac.cn/gsa-human/submit/hra/subHRA021764/detail) ( [https://ngdc.cncb.ac.cn/gsa-human/browse/HRA014397] ). The mass spectrometry proteomics data have been deposited to the ProteomeXchange Consortium (https://proteomecentral.proteomexchange.org) via the iProX partner repository [63,64] with the dataset identifier PXD070868.
