# Supplementary figures and images for: Identification of candidate microRNA biomarkers of endometriosis in different bodily fluids
Source: Sci Rep. 2026 Jan 25;16:6218. doi: 10.1038/s41598-026-37277-5 (PMC12905408; doi:10.1038/s41598-026-37277-5)

Reads mapped to miRNAs

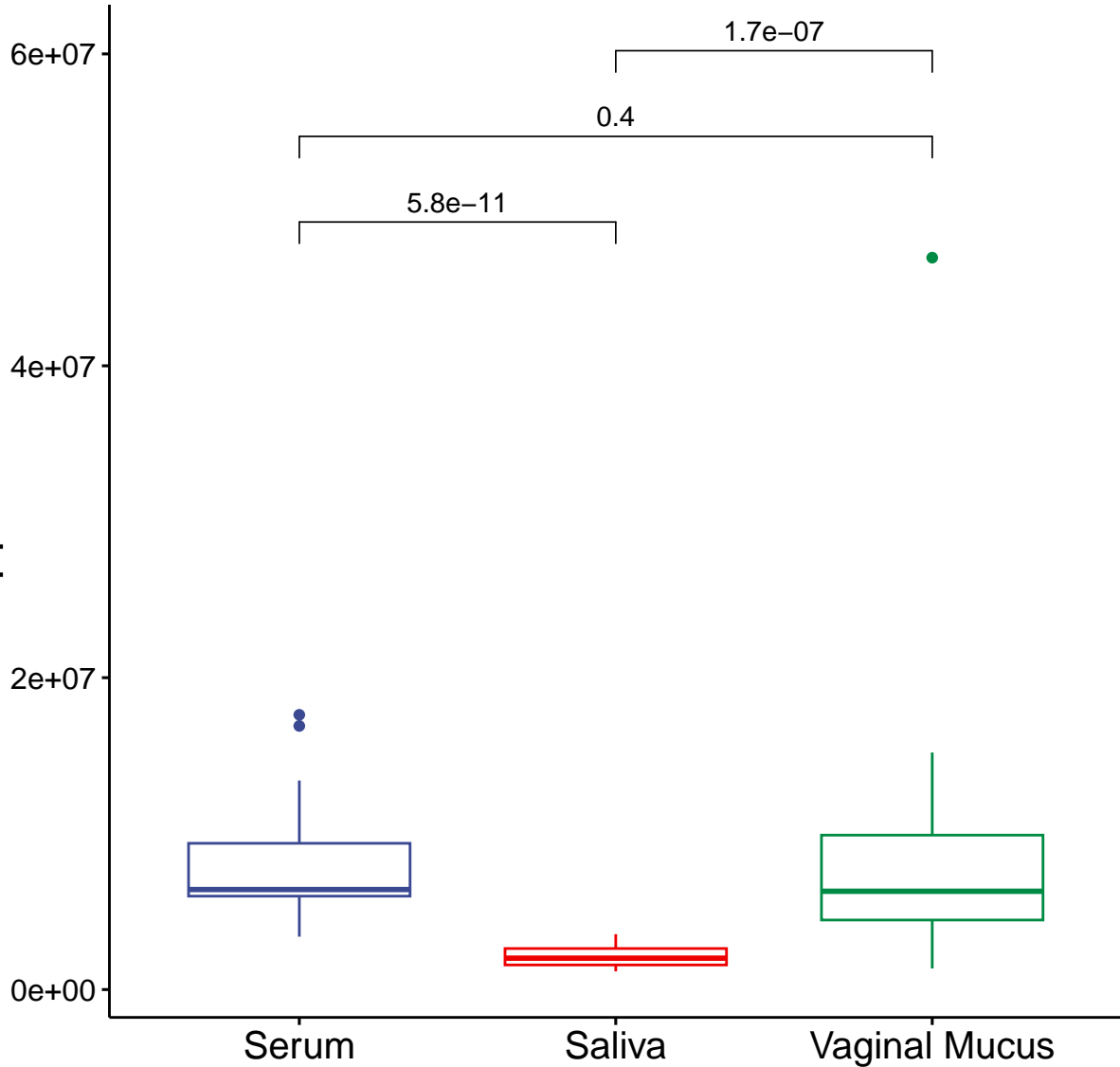

Supplement: Supplementary file 3 — Supplementary Material 3 [file 41598_2026_37277_MOESM3_ESM.pdf]
