# Supplementary material for: Identification of candidate microRNA biomarkers of endometriosis in different bodily fluids
Source: Sci Rep. 2026 Jan 25;16:6218. doi: 10.1038/s41598-026-37277-5 (PMC12905408; doi:10.1038/s41598-026-37277-5)
Supplement: Supplementary file 4 — Supplementary Material 4 [file 41598_2026_37277_MOESM4_ESM.docx]

**Supplementary Materials**

Table S1. Number of total reads and unique miRNAs per sample group.

| **Sample type** | **Total Reads (M)** | **Unique miRNAs** | **Mapping rates (×10⁻⁵)** |
| --- | --- | --- | --- |
| Vaginal mucus (mean±SD) | 31.54 ± 10.31 | 491.65 ±201.15 | 1.58 ± 0.44 |
| Saliva (mean±SD) | 34.01 ± 14.19 | 245.65 ± 72.95 | 0.82 ± 0.39 |
| Serum (mean±SD) | 28.21 ± 6.63 | 668.05 ± 80.24 | 2.49 ± 0.60 |

Figure S1. Read counts mapped to miRNAs.


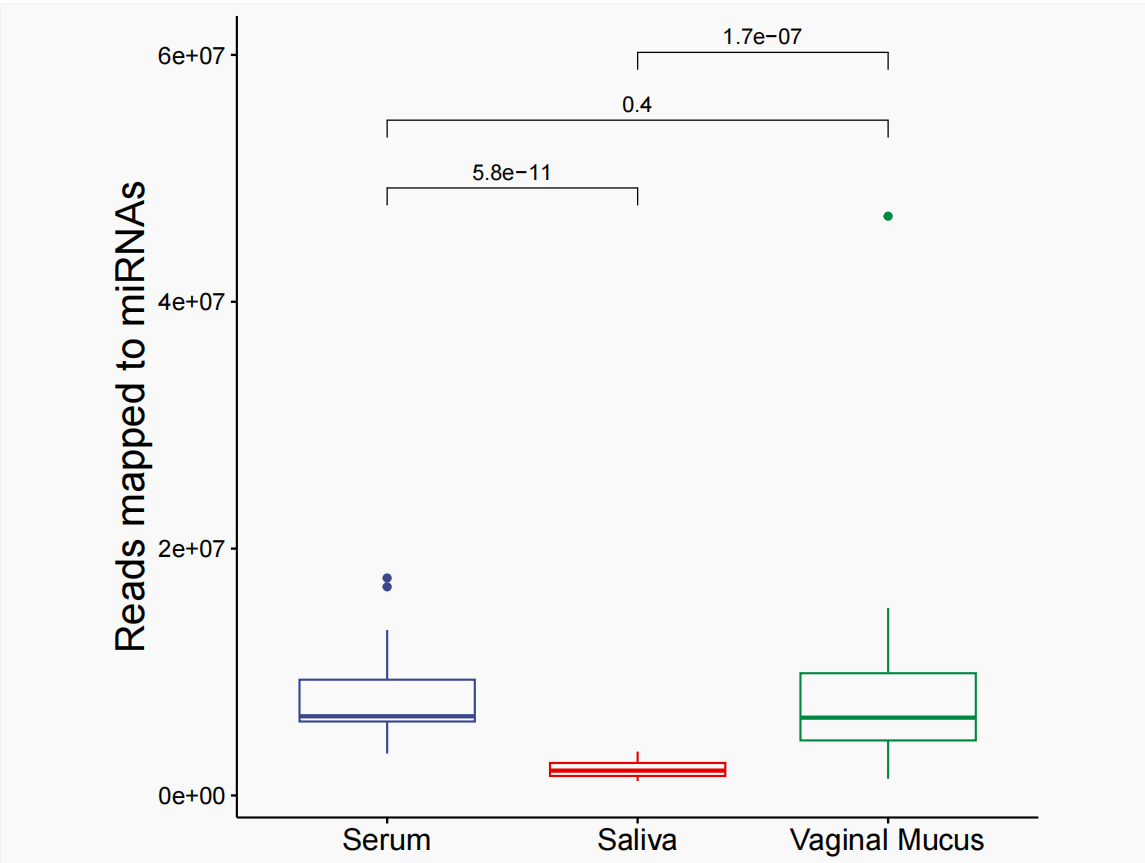


Figure S2. MiRNA expression profiles per sample.


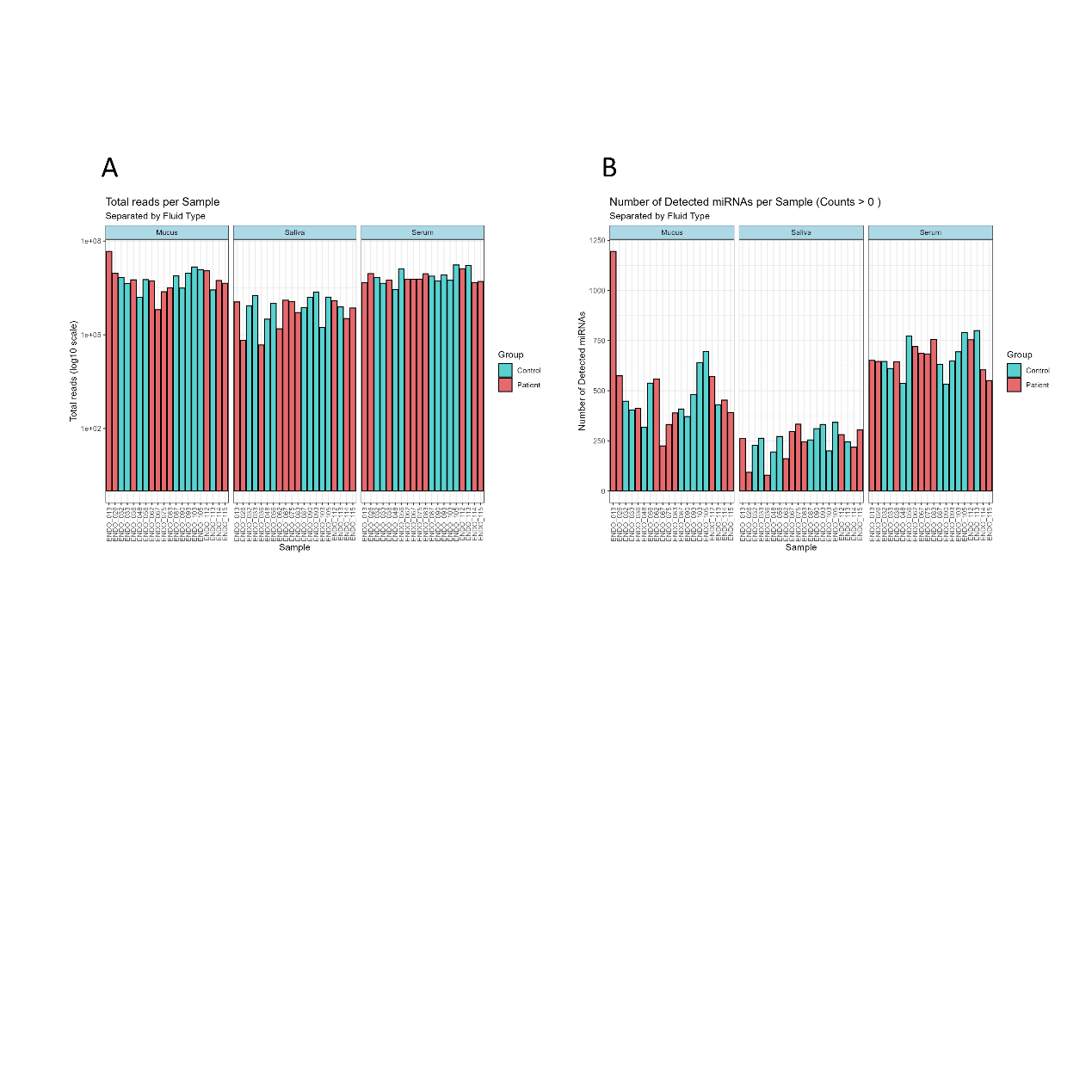


A, Bar plot illustrating the total number of raw reads (library size) for each individual sample, presented on a base-10 logarithmic scale. B, Bar plot showing the number of unique miRNAs detected in each sample, defined as miRNAs with a read count greater than 0.
